# Multiple signals in anterior cingulate cortex

**DOI:** 10.1016/j.conb.2015.12.007

**Published:** 2016-04

**Authors:** N Kolling, TEJ Behrens, MK Wittmann, MFS Rushworth

**Affiliations:** 1 Department of Experimental Psychology, University of Oxford, Oxford, UK; 2 Centre for Functional MRI of the Brain (FMRIB), Nuffield Department of Clinical Neurosciences, John Radcliffe Hospital, University of Oxford, UK

## Abstract

Activity in anterior cingulate cortex (ACC) has been linked both to commitment to a course of action, even when it is associated with costs, and to exploring or searching for alternative courses of action. Here we review evidence that this is due to the presence of multiple signals in ACC reflecting the updating of beliefs and internal models of the environment and encoding aspects of choice value, including the average value of choices afforded by the environment (‘search value’). We contrast this evidence with the influential view that ACC activity is better described as reflecting task difficulty. A consideration of cortical neural network properties explains why ACC may carry such signals and also exhibit sensitivity to task difficulty.

## Highlights

• There are multiple signals in anterior cingulate cortex (ACC).
• ACC activity reflects value of behavioural change even after controlling for difficulty.
• ACC activity reflects updating of internal models even after controlling for difficulty.

**Current Opinion in Neurobiology** 2016, **37**:36–43

This review comes from a themed issue on **Neurobiology of cognitive behavior**

Edited by **Alla Karpova** and **Roozbeh Kiani**

For a complete overview see the Issue and the Editorial

Available online 8th January 2016

**http://dx.doi.org/10.1016/j.conb.2015.12.007**

0959-4388/© 2016 The Authors. Published by Elsevier Ltd. This is an open access article under the CC BY license (http://creativecommons.org/licenses/by/4.0/).

When humans and other animals take a course of action they usually do so because they believe the benefits of doing so will outweigh the costs. There is an evolving understanding of the mechanisms underlying evaluation of one well-defined choice against another that have been linked to ventromedial, orbital prefrontal, and intraparietal sulcal cortex [1, 2••, 3, 4••, 5]. There are also, however, times when animals decide whether it is worth acting at all or evaluate whether it is worth continuing to engage in the current behaviour or to explore alternatives. This distinct pattern of decision-making is linked to ACC; ACC manipulations affect the ability of animals to initiate any action at all [6], weigh up the costs and benefits of actions [7, 8••], switch between actions as their values change [9, 10••], or explore alternative choices [11^••^]. A series of recent studies have demonstrated the presence of activity changes in ACC that correspond to the types of signals that would be needed to guide such behaviour; these signals encode the values of actions [7, 12••, 13, 14, 15, 16••, 17••, 18••], the average value of alternative courses of action in the environment (‘search value’) as opposed to the current or default course of action [19, 20, 21], exploration and evaluation of hypotheses about the best course of action to take [22, 23••, 24], and reflect updating of decision-makers’ beliefs and internal models of their environments [25, 26]. Not only are such signals found in ACC but they are weak or absent in regions such as orbitofrontal and ventromedial prefrontal cortex that carry other value signals [19, 20, 22].

In addition, however, ACC has also been linked to ‘conflict monitoring’ — the process of detecting when two competing choices might be made during a difficult task [27]. Detecting response conflict and task difficulty is important if mistakes are to be averted. Recently it has been argued that ACC activity interpreted as reflecting value signals has been confounded with difficulty and so it has been argued that such ACC activity is more parsimoniously interpreted as simply reflecting task difficulty [28]. Here we review evidence, first, that value signals and, second, model update signals can be separated from any effect difficulty exerts on ACC activity.

For example, a recent study [19] investigated how people decide whether to explore a set of alternative choices or stick with the opportunity to make a ‘default’ choice. The value of exploring was encoded by a ‘search value’ signal in ACC indexing the average value of the set of alternative choices that might be taken. In addition to *search value*, ACC activity was also influenced, in a negative fashion, by *engage value* (the value of the default option) and *costs* incurred by searching. This pattern of positive and negative modulations is suggestive of a comparison process taking place within ACC that could inform decisions about whether or not to explore, or ‘forage’ amongst, the alternatives.

**Figure 1:**
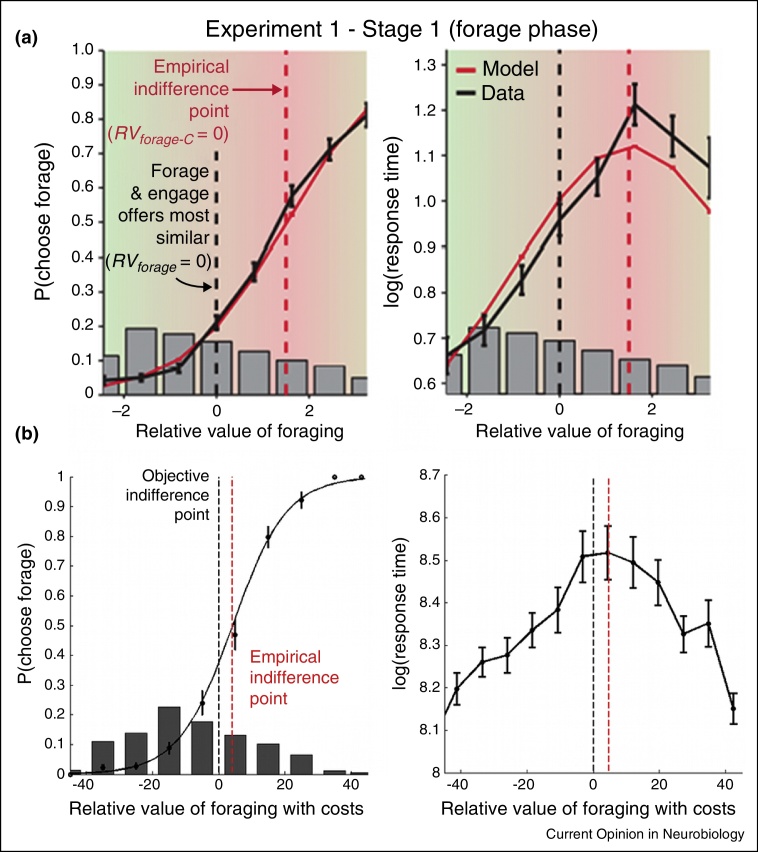
**Avoiding confounds between value and difficulty. (a)** Foraging frequency (left) and difficulty, as indexed by log(RT) (right), as a function of RVF in an experiment claiming value signals and difficulty have been confounded in ACC [28]. The black line indicates behavioural data and the red one the corresponding model fit. The grey bars are the sample sizes and the dotted lines are the two indifference points (red = subjective or empirical and black = objective indifference point, i.e. where the value of searching and engaging are objectively equal; ‘RV_forage_ = 0’). The participants tended not to forage and to be inaccurate. For example, foraging frequency barely reaches 80% even on the right hand side of the left panel and the participants’ empirical indifference points were far from the objective indifference point. **(b)** After adequate task training and instruction in a version of the task employing a balanced and evenly sampled range of search and engage values in which decisions are non-trivial and require value comparison [19] several features of the experiment, participant performance, and data are notable: (i) participants balance all the factors that should influence decision-making in an approximately rational manner and the point of empirical indifference is close to the objective indifference point meaning that ii) data are sampled from both left and right of decision space ensuring foraging values and difficulty decorrelation; iii) Log(RT) decreases either side of the empirical indifference point in an approximately similar manner confirming foraging values and log(RT) are not correlated. Foraging decisions plotted (similar format to a) as a function of RVF (based on all three variables that should influence behaviour: search value, engage value, and an additional factor related to the cost of foraging). Adapted from [19, 28].

Figure 1a, however, summarizes how difficulty might be confounded with the difference between *search* and *engage value* — a quantity sometimes referred to as the ‘relative value of foraging’ or RVF [28]. The probability of behavioural change — searching as opposed to ‘engaging’ with the current default — is plotted on the ordinate as a function of RVF. A confound between RVF and difficulty arises if subjects are biased to take the default. Even if the experiment examines decisions equally on either side of the objective indifference point — the point at which searching and engaging objectively have the same value — it is still possible that the sampling is unequal with respect to the subjective or empirical indifference point — the point at which a given participant has no preference between the options. The confound arises because decisions close to the subjective indifference point are the most difficult to take [for example, they are associated with long reaction times (RTs)]. If participants are very biased to nearly always take the default option then RVF and difficulty both increase together across much of the decision space.

**Figure 2:**
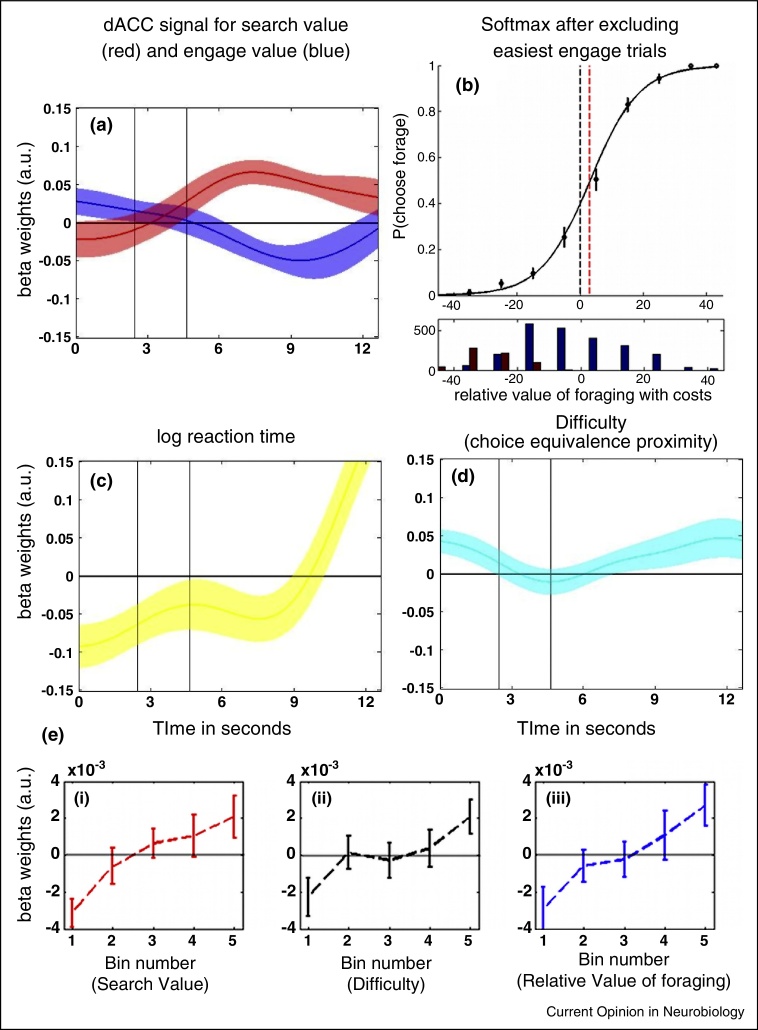
**Search value has an early and sustained effect on ACC activity, engage value impacts on ACC slightly later, and difficulty effects occur even later in the trial. (a)** General linear model (GLM) timecourse analysis ACC activity demonstrates effects of both search value (red) and engage value (blue). Note that RVF is a combination of search value and engage value. The results remain the same regardless of whether the regression included all the data from [19] and regressors indexing the cost of taking a foraging choice, difficulty, and/or logRT. They remain the same even if, to further guard against any possibility of a confound, the analysis focused on the data that best discriminates between search value and difficulty. This can be achieved by focusing on a subset of the data. To ensure no correlation between RVF or search value and difficulty or log(RT) the easiest engage trials where *p*(forage) < 0.02 (lower panel) can be removed. The numbers of samples included are shown in blue in the lower panel while the excluded trials are shown in red. Forage frequency in the remaining trials is shown in the upper panel. The effect of log(RT) **(c)** and difficulty **(d)** appear late in the trial. Statistical significance of signals can be assessed by convolving the time-course of their beta-weights with a hemodynamic function (*μ* = 6 and *σ* = 3; to average the beta-weights of each contrast and every person separately). Search value had a significant effect on ACC (*p* ≤ 0.001 in all cases). Difficulty had little impact on ACC activity as estimated using a standard hemodynamic function time-locked to the start of the trial or response cue onset, but the effect of difficulty and RT increased later in the trial. **(e)** HRF convolved average BOLD signal in ACC binned according to different parameters. When ACC activity is examined late in the trial period it can be seen that it increases with search value (e, i), difficulty (e, ii) and RVF (e, iii). When the same analysis is conducted earlier in the trial then only search value and RVF effects are apparent. All bins are equally sized for every participant and included at least 32 trials. Error bars are the standard error of the individual effects for each bin. Adapted from [19].

Experiments addressing this criticism must contain certain obvious features. First, a broad and evenly distributed range of search and engage value must be tested. However, at the same time, it is crucial that decisions are not trivially easy and that some value comparison occurs on each trial. Second, it is imperative that participants make decisions that really are guided by option values and do not always simply engage with the default option. One way of ensuring this is simply to provide adequate task training and instruction prior to scanning. If this is done then subjects make rational value-guided decisions and therefore subjective and objective indifference points are close (Figure 1c) and difficulty/value confounds disappear; many decisions are examined in decision space to the right of the subjective indifference point where RVF and difficulty are not positively correlated. Third, when analysing the data, rather than examining the neural correlates of the aggregate of decision variables — RVF — it is advisable to focus on the component values that determine RVF: search value, engage value, and costs. These component values are more easily dissociated from difficulty. Employing these principles Kolling and colleagues [19] reduced the shared variance between search value, engage value, and difficulty to 2% so that the neural correlates of each could be separately identified (Figure 2). Now it is clear that ACC activity reflects search value shortly followed by engage value although towards the end of the decision period some variance in ACC activity is accounted for by difficulty and RT. Parallels can be drawn with recordings made in other brain areas concerned with value-guided decision making such as the intraparietal sulcus [29]; initially activity in intraparietal neurons reflects saccade value but then it transitions to reflect action related factors.

Such a pattern of results suggests ACC is a neural network in which decisions to explore or not are taken; activity is affected by a search value signal (apparent throughout much of the trial period) but that the network takes longer to make decisions using this signal and others when they are difficult [30] (and therefore some variance in dACC activity at the end of the decision period is accounted for by difficulty and RT). Biophysically plausible neural networks have been proposed [31] in which pools of neurons are active in proportion to the evidence favouring particular choices. If the representation of search value in ACC takes this form then the network activity should reflect both search value and difficulty. In fact, the prediction is that the impact of search value should scale with difficulty. Although it might be difficult to assess such a precise hypothesis with fMRI such considerations suggest that conducting experiments with decisions involving extremely high search values may be unwise [28]; when decision difficulty is very low the network may resolve the decision and enter an attractor state so quickly that it will be difficult to see any effect of search value. In other words, exclusive sensitivity to search value, and not difficulty too, is not a prediction for a search value sensitive decision circuit but instead sensitivity to both search value and difficulty is expected. In the future, careful neurophysiological measurements will be essential for testing such potential mechanisms at the neuronal level, disentangling how aggregate measures such as the BOLD signal are derived from actual neural network operations.

**Figure 3:**
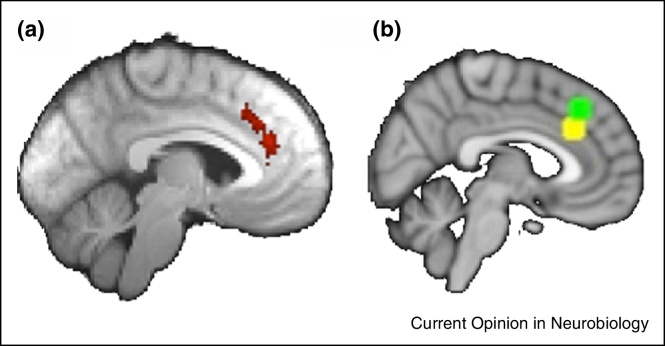
**Value effects are prominent in ACC proper while difficulty effects are more prominent in more dorsal areas in or near pre-SMA**. **(a)** Whole brain cluster-corrected effect of search value (peak *z* = 3.2 at Montreal Neurological Institute (MNI) coordinates [−4, 36, 26] in ACC after controlling for difficulty and log(RT) using data shown in Figure 1c and Figure 2. **(b)** Previous reports [19] of search value related activity emphasized a similar ACC location [MNI, 4, 28, 30, yellow]. In a study [28] emphasizing difficulty, effects were in or just anterior to pre-SMA (MNI, 4, 32, 42, green). The one exception, reported in the Supplementary Analysis was at a point intermediate between the yellow and green areas that is probably within the border of ACC [MNI, 6, 28, 34]. Adapted from [19].

Furthermore, ACC is sometimes co-activated with adjacent medial frontal brain areas [26] and so an important consideration when drawing conclusions about ACC is to ensure that neural activity that is recorded really is drawn from ACC rather than adjacent medial frontal areas. After controlling for difficulty, search value effects are most prominent in ACC itself (Figure 3a) but task difficulty effects lie in more dorsal areas in or anterior to the pre-supplementary motor area (Figure 3b).

Humans and other animals should change from the behaviour they are currently engaged in and explore alternative courses of action not just when they have a sense of the value of those alternatives but also when they realise the environment is changing. ACC activity is also prominent when events suggest that a decision-maker's internal model of their environment should be updated [11••, 25, 26]. By definition, surprising events are ones that were not predicted by the decision-maker's current model of their environment. They are, therefore, frequently the events that indicate the need for model updating. At the same time, however, surprising events are often events to which responses are made more slowly (longer RTs) and with greater difficulty because the response, or the stimulus eliciting it, was unanticipated. Does ACC activity at the time of model updating simply reflect task difficulty — the difficulty of responding when internal model updating occurs? Or does it activate when internal models have to change?

**Figure 4:**
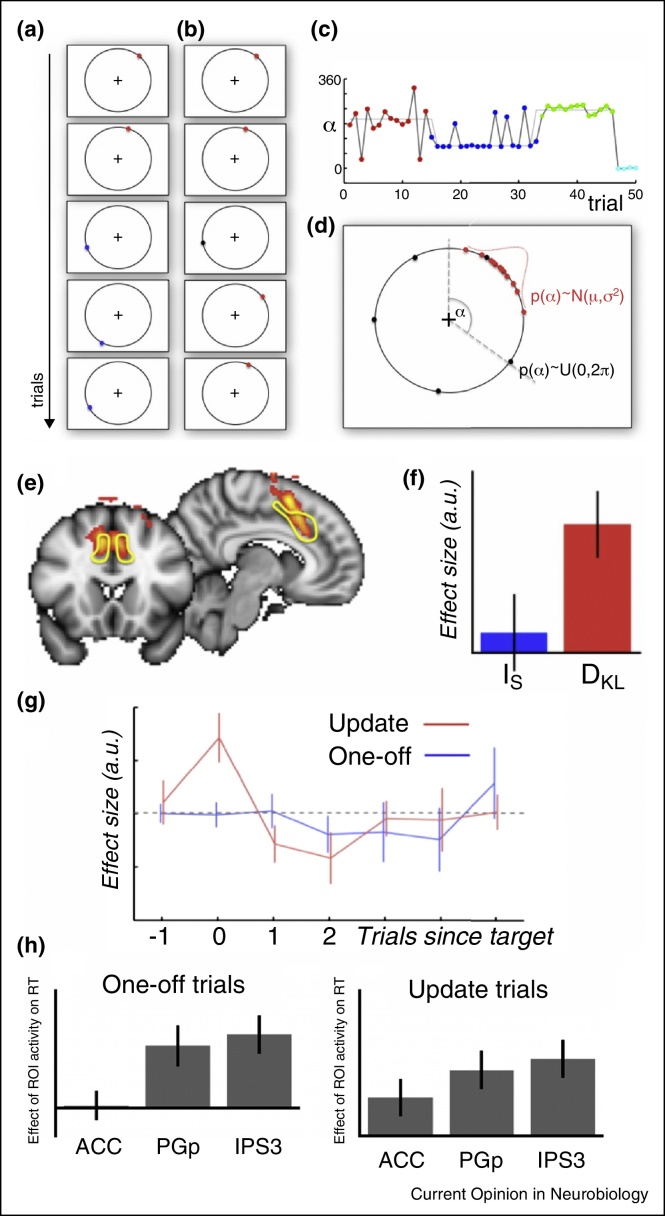
**ACC is active when internal models are updated not just when task difficulty increases because surprising events occur.** On each trial of a saccade planning task, participants began by fixating a central cross. A target (coloured dot) appeared on a circular perimeter. Its location was predictable because target locations were similar over runs. However, two types of unexpected targets occurred. **(a)** On model update trials the new dot location was indicative of future dot locations. To signal those trials, the dot had a different colour. In the example, before the update, trials had red dots in the upper right and the model update trial had a blue dot in the lower right. **(b)** This was not the case on surprise only trials. There the dot colour was grey and future targets reverted to the original distribution (in the example to the upper right) **(c)** Plot of target locations (angle *α* from vertical) over 150 trials. Different coloured targets are from different runs. One-off targets are shown in grey. **(d)** Distribution of target locations within a run is a combination of a circular Gaussian, shown in red, and a uniform distribution, shown in black, from which one-off trials were drawn. **(e)** Whole-brain cluster-corrected fMRI analysis indicated a region spanning ACC and adjacent pre-supplementary motor area was the only area in which there was a significant effect of model updating (contrast shows all voxels with a parametric effect of *D*_KL_). The ROI denoted by the yellow line is the ACC region of interest analysed in panels E and F. **(f)** Mean effect size for surprise (*I*_S_) and updating (*D*_KL_) in the ACC ROI (error bars are SEM). **(g)** Raw activity in the ACC ROI plotted as a function of trial-in-run (0 on abscissa indicates model update or surprise trial, while trials 1, 2, 3, etc., are the trials following the model update or surprise trial. (e) At last, there are regions other than the dACC that are more active as a simple function of the reaction time, which is mostly a function of the difficulty of responding, similarly in one off and update trials (left and right panel).). Adapted from [26].

A recent study tested exactly this distinction [26] (Figure 4) while controlling for response confounds and showed that model update signals can indeed be dissociated from task difficulty effects. Human participants made saccades to targets (coloured dots) that, on each trial, appeared on a circular perimeter surrounding a fixation point. The dots’ locations were usually predictable because they were similar over runs of 10–20 trials but two types of unexpected event occurred. On *model update* trials (Figure 4a) the dot appeared in an unexpected location and its new colour indicated that future dots were likely to appear nearby on the circle's periphery. However, on *surprise only* trials (Figure 4b), dots appearing in white in a surprising location indicated one-off events and no need for participants to update their internal model of where future dots would appear. The difficulty of responding on any trial reflects the surprise associated with a particular stimulus value, *α*, and is characterized in Information Theory by its Shannon information *I*_S_(*α*):

(1) $$I_{S}(\alpha )=-\text{log}\;p(\alpha \left|\text{Prior}\right))$$

where *p*(*α*|prior) is the prior probability that the observation *α* would be made, given the brain's internal model just before the data point was observed. Therefore the Shannon information captures how unexpected or unlikely a particular observation is, given the internal model and is directly related to the difficulty of the trial. In contrast, updating of the internal model is captured by the Kullback–Leibler divergence (*D*_KL_) between the posterior and the prior:

(2) $$D_{KL}\left(\text{post}∥\text{prior})\right)=\sum_{\alpha }p\left(\alpha \left|\text{prior}\right)\right)\left[\log\;p\left(\alpha \left|\text{prior}\right)\right)-\log\;p\left(\alpha \left|\text{post}\right)\right)\right]$$

where *p*(*α*|prior) is the probability that the observation *α* would be made, given the model just before *α* was observed, and *p*(*α*|post) is the same quantity, given the updated model just after *α* was observed. *D*_KL_ is the probability-weighted *average* change in Shannon information across *all possible stimuli* as a consequence of updating the model.

Although RTs increased on both model update and surprise trials ACC was preferentially engaged on model update trials. Moreover ACC activity covaried with the model updating parameter, *D*_KL_, but not surprise *I*_S_ (Figure 4e and f), despite *I*_S_'s relationship to difficulty. Although parietal regions were active as a function of response selection difficulty as indexed by RT, ACC was not (Figure 4h). Other studies similarly suggest ACC is activated when there is a need to update the task model even in the absence of any response selection difficulty (because no response is required at all) [32]. Model updating-related activity in ACC is, therefore, linked to behavioural flexibility and change and not simply response selection difficulty. This role of the ACC may underlie its activation during proactive control and error correction. It is possible that ACC activity in other experiments may have a similar role [21, 33••].

In summary, ACC carries multiple signals. ACC activity reflects both search value and the updating of internal models of the environment. In both cases, and in other reports [20, 34, 35], ACC is linked to behavioural change, invigoration of new responses, novel response strategies, and exploration. We have conceptualized search value as the average value of choices that might be taken in an environment but it could take many other forms depending on context. We and others have argued that some of these signals may have arisen in the context of the foraging choices that animals make as they decide to leave one foraging patch to explore another [15, 19, 20, 36, 37]. Advantages of this approach are that it situates ACC function within the context of a behaviour for which there has been substantial evolutionary pressure and it suggests ways of optimal modelling of both behaviour and neural activity. Similar processes are likely to underlie human behaviours such as task switching. Such a perspective holds great promise for making novel predictions about behaviour and neural mechanisms in a principled fashion.

Two regions within ACC, dorsal ACC (dACC) and perigenual ACC (pgACC) [19, 20, 38••, 39••], carry related signals. Both areas are found in humans and macaques; each area has a distinctive pattern of interaction with wider brain circuits that is similar across species [40, 41••]. Similar areas are also present in rodents and again they mediate related aspects of behaviour [8••, 11••, 25, 42]. Indeed, when a decision-maker has updated its internal model or is about to pursue an alternative course of action then it may be necessary to exert careful control over which actions are selected next. However, the same is true even when one manages to resist the attractions of an alternative course of action [43] or when attention has lapsed or errors have been made. In all these situations it is necessary to exert greater cognitive control and this may be brought about by interactions between ACC and lateral prefrontal cortex [16••, 23••, 24, 44••, 45, 46, 47, 48, 49••].

## Conflict of interest

We have no conflicts of interest.

## References and recommended reading

Papers of particular interest, published within the period of review, have been highlighted as:

• of special interest
•• of outstanding interest
